# Supplementary material for: Role of Rifaximin in the Prognosis of Critically Ill Patients with Liver Cirrhosis
Source: Antibiotics (Basel). 2025 Mar 10;14(3):287. doi: 10.3390/antibiotics14030287 (PMC11939653; doi:10.3390/antibiotics14030287)
Supplement: Supplementary file 1 [file antibiotics-14-00287-s001.zip › antibiotics-3508083-supplementary.pdf]

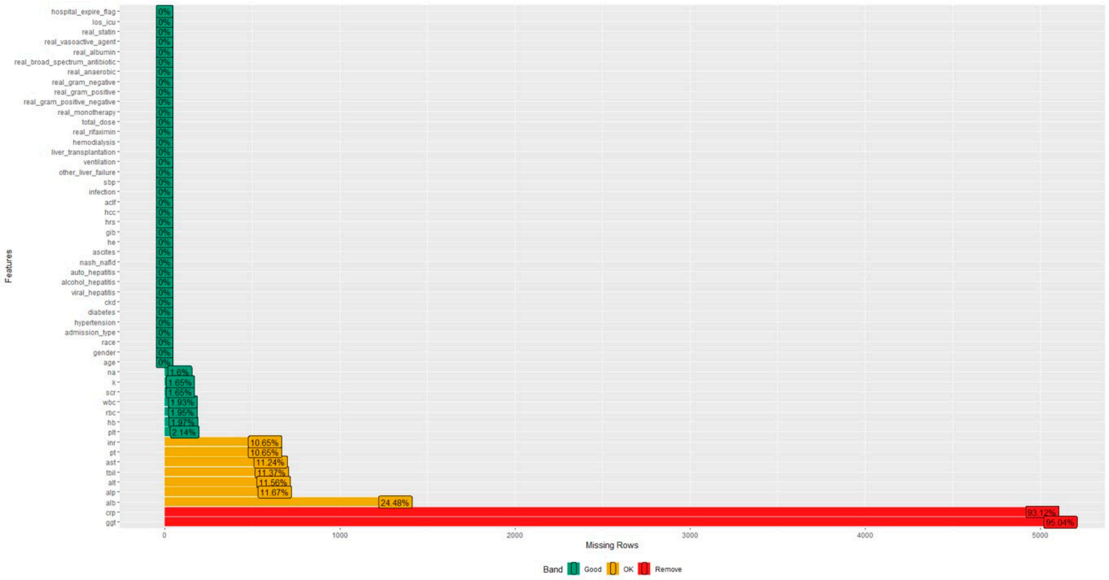

Supplementary Figure S1. Percent missing values of baseline information at hospital admission. HE, hepatic encephalopathy; SBP, spontaneous bacterial peritonitis; HRS, hepatorenal syndrome; ACLF, acute-on-chronic liver failure; NAFLD, non-alcoholic fatty liver disease; GIB, gastrointestinal bleeding; HCC, hepatocellular carcinoma; Hb, hemoglobin; WBC, white blood cell; PLT, platelet, TBIL, total bilirubin; ALT, alanine aminotransferase; AST, aspartate aminotransferase; ALB, serum albumin; ALP, alkaline phosphatase; GGT, glutamyl transpeptidase; CRP, C-reaction protein; Scr, serum creatinine; Na, sodium; K, potassium; PT, prothrombin time; INR, international normalized ratio.

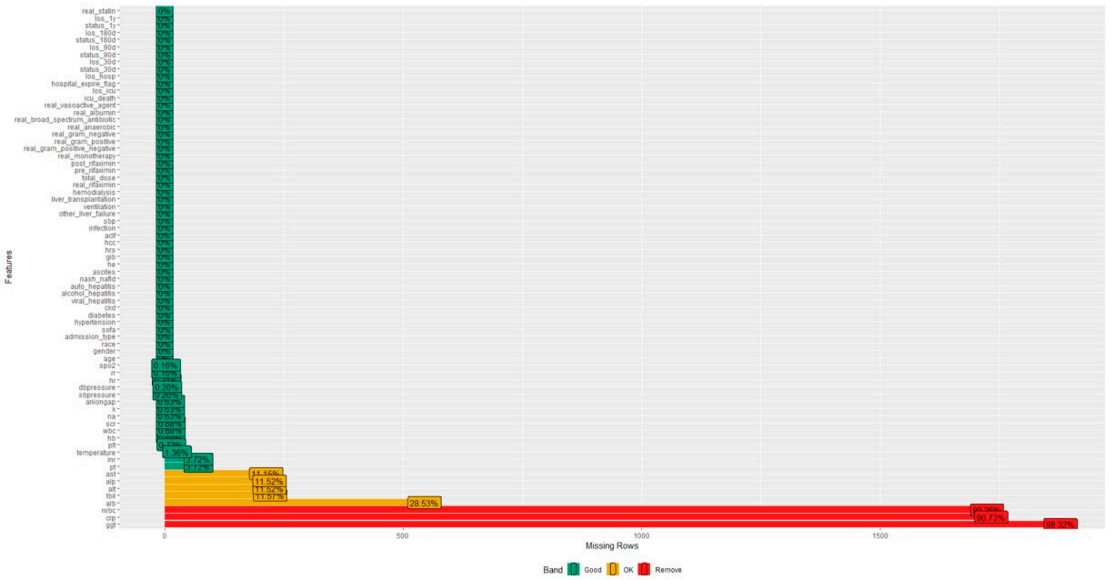

Supplementary Figure S2. Percent missing values of baseline information at ICU admission. HE, hepatic encephalopathy; SBP, spontaneous bacterial peritonitis; HRS, hepatorenal syndrome; ACLF, acute-on-chronic liver failure; NAFLD, non-alcoholic fatty liver disease; GIB, gastrointestinal bleeding; HCC, hepatocellular carcinoma; Hb, hemoglobin; WBC, white blood cell; PLT, platelet, TBIL, total bilirubin; ALT, alanine aminotransferase; AST, aspartate aminotransferase; ALB, serum albumin; ALP, alkaline phosphatase; GGT, glutamyl transpeptidase; CRP, C-reaction protein; Scr, serum creatinine; Na, sodium; K, potassium; PT, prothrombin time; INR, international normalized ratio.

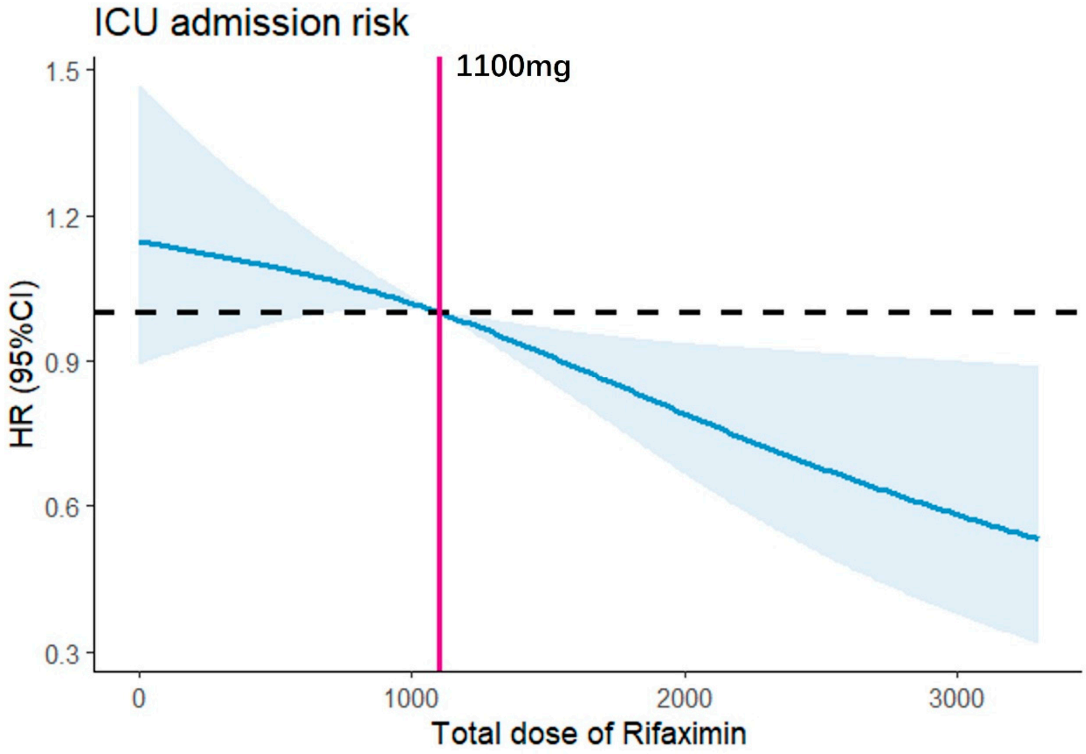

Supplementary Figure S3. Restricted cubic spline regression analysis of the relationship between total dose of rifaximin and the risk of ICU admission. ICU, intensive care unit; HR, hazard ratios; CI, confidence intervals.

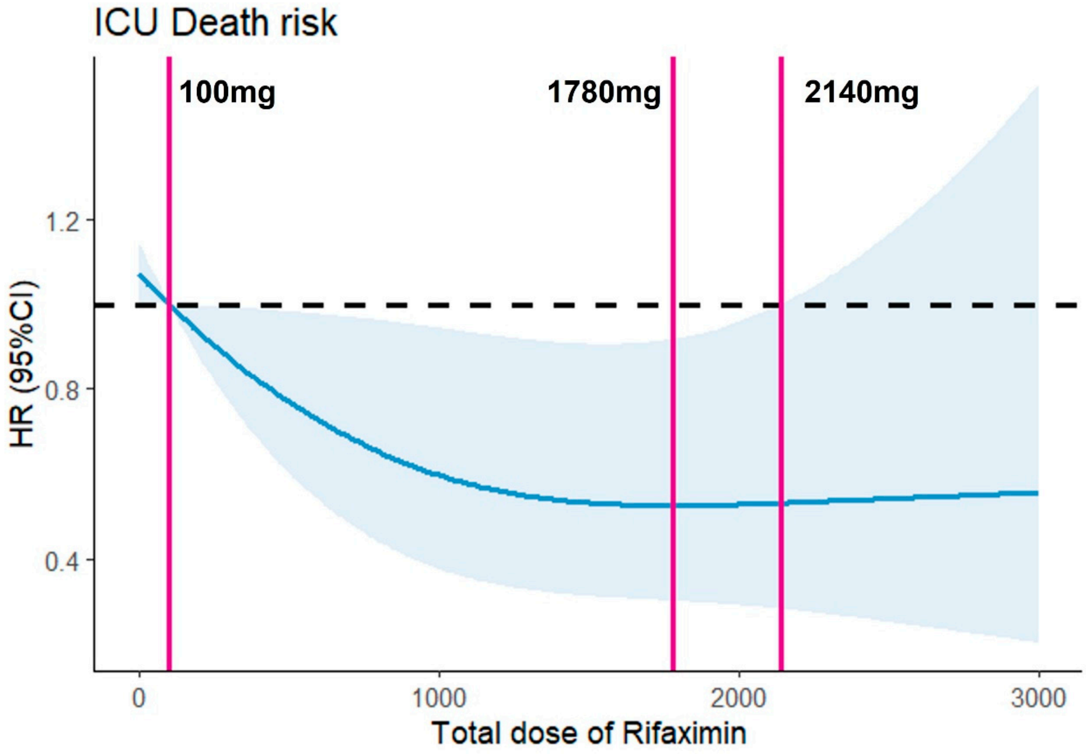

Supplementary Figure S4. Restricted cubic spline regression analysis the relationship between total dose of rifaximin and the risk of ICU death. ICU, intensive care unit; HR, hazard ratios; CI, confidence intervals.

Variable	Count	Percent	HR (95% CI)	P value	P for interaction
Overall	986	100	0.12 (0.03 to 0.43)	0.001	
Gender					0.837
F	344	34.9	0.14 (0.02 to 1.27)	0.08	
M	642	65.1	0.10 (0.02 to 0.52)	0.006	
CKD					0.997
No	846	85.8	0.17 (0.04 to 0.65)	0.009	
Yes	140	14.2	0.07 (0.00 to 0.82)	0.032	
Ascites					0.694
No	474	48.1	0.16 (0.03 to 0.86)	0.033	
Yes	512	51.9	0.12 (0.01 to 0.99)	0.048	
GIB					0.998
No	977	99.1	0.13 (0.04 to 0.47)	0.002	
Yes	9	0.9	0.17 (0.00 to 3.12)	0.228	
HE					0.997
No	873	88.5	0.11 (0.03 to 0.43)	0.001	
Yes	113	11.5	0.18 (0.00 to 2.88)	0.23	
HRS					0.738
No	934	94.7	0.10 (0.02 to 0.51)	0.005	
Yes	52	5.3	0.11 (0.01 to 1.13)	0.063	
SBP					0.758
No	916	92.9	0.10 (0.02 to 0.49)	0.004	
Yes	70	7.1	0.17 (0.00 to 6.12)	0.331	
HCC					0.998
No	955	96.9	0.12 (0.03 to 0.45)	0.002	
Yes	31	3.1	0.30 (0.00 to 5.55)	0.421	
ACLF					1
No	960	97.4	0.10 (0.03 to 0.37)	0.001	
Yes	26	2.6	1.00 (1.00 to 1.00)		
Broad spectrum antibiotic					0.511
No	822	83.4	0.04 (0.00 to 0.47)	0.01	
Yes	164	16.6	0.09 (0.01 to 0.74)	0.025	

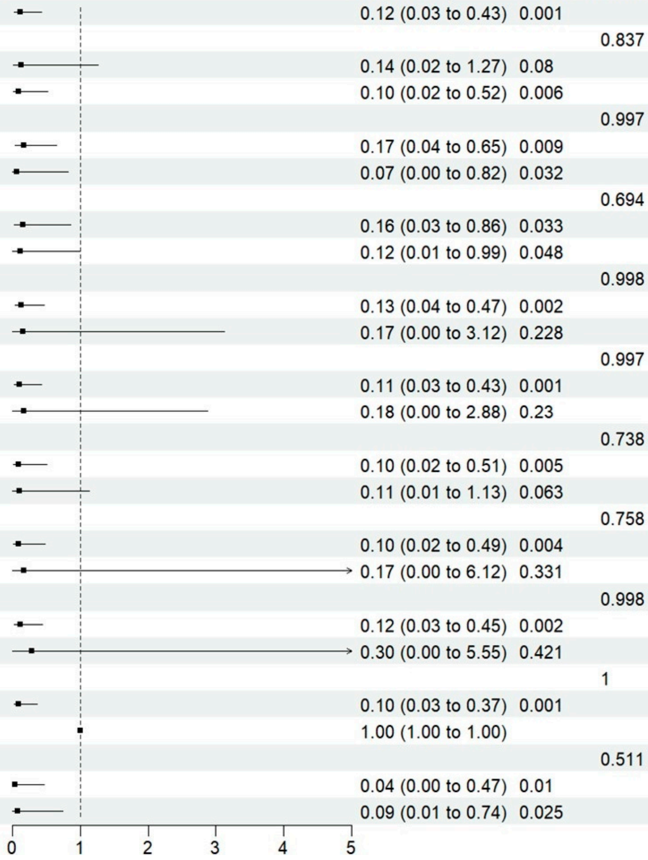

Supplementary Figure S5. Subgroup analysis of the effect of rifaximin on the risk of in-hospital death. F, female; M, male; CKD, chronic kidney disease; HE, hepatic encephalopathy; SBP, spontaneous bacterial peritonitis; HRS, hepatorenal syndrome; ACLF, acute-on-chronic liver failure; GIB, gastrointestinal bleeding; HCC, hepatocellular carcinoma.

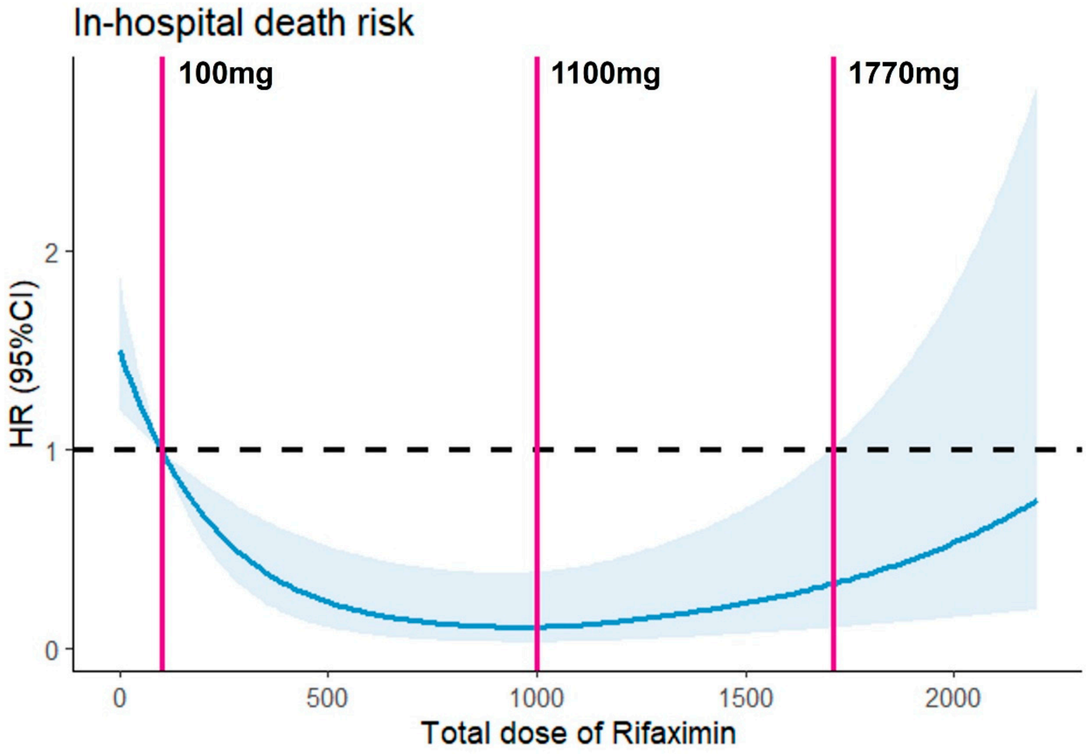

Supplementary Figure S6. Restricted cubic spline regression analysis the relationship between total dose of rifaximin and the risk of in-hospital death. HR, hazard ratios; CI, confidence intervals.

Variable	Count	Percent	HR (95% CI)	P value	P for interaction
Overall	986	100	0.91 (0.66 to 1.24)	0.541	
Gender					0.463
F	344	34.9	0.76 (0.44 to 1.31)	0.328	
M	642	65.1	0.99 (0.67 to 1.47)	0.975	
CKD					0.145
No	846	85.8	0.79 (0.55 to 1.13)	0.195	
Yes	140	14.2	1.55 (0.76 to 3.19)	0.231	
Ascites					0.558
No	474	48.1	1.03 (0.58 to 1.85)	0.919	
Yes	512	51.9	0.85 (0.58 to 1.24)	0.388	
GIB					0.684
No	977	99.1	0.91 (0.66 to 1.25)	0.559	
Yes	9	0.9	0.34 (0.00 to 7.69)	0.475	
HE					0.776
No	873	88.5	0.89 (0.63 to 1.25)	0.499	
Yes	113	11.5	1.02 (0.41 to 2.58)	0.959	
HRS					0.617
No	934	94.7	0.87 (0.61 to 1.23)	0.423	
Yes	52	5.3	1.08 (0.48 to 2.44)	0.856	
SBP					0.112
No	916	92.9	0.83 (0.60 to 1.16)	0.281	
Yes	70	7.1	3.01 (0.85 to 10.58)	0.086	
HCC					0.359
No	955	96.9	0.93 (0.67 to 1.30)	0.686	
Yes	31	3.1	0.63 (0.20 to 1.98)	0.432	
ACLF					0.198
No	960	97.4	0.87 (0.62 to 1.20)	0.394	
Yes	26	2.6	2.79 (0.66 to 11.74)	0.162	
Broad_spectrum_antibiotic					0.216
No	822	83.4	1.01 (0.71 to 1.44)	0.96	
Yes	164	16.6	0.60 (0.29 to 1.24)	0.168	

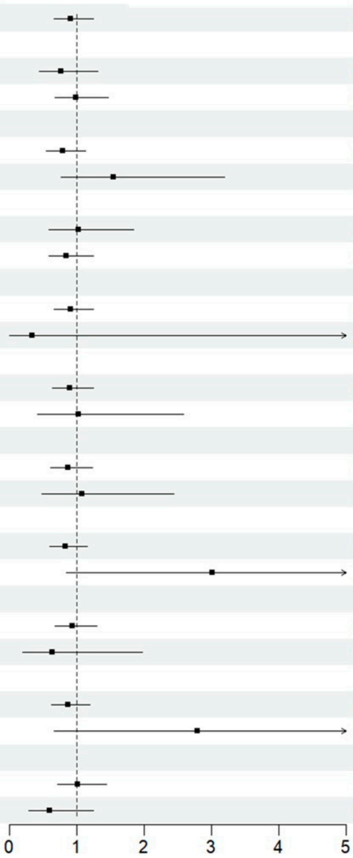

Supplementary Figure S7. Subgroup analysis of the effect of rifaximin on the risk of 90-day death. F, female; M, male; CKD, chronic kidney disease; HE, hepatic encephalopathy; SBP, spontaneous bacterial peritonitis; HRS, hepatorenal syndrome; ACLF, acute-on-chronic liver failure; GIB, gastrointestinal bleeding; HCC, hepatocellular carcinoma.

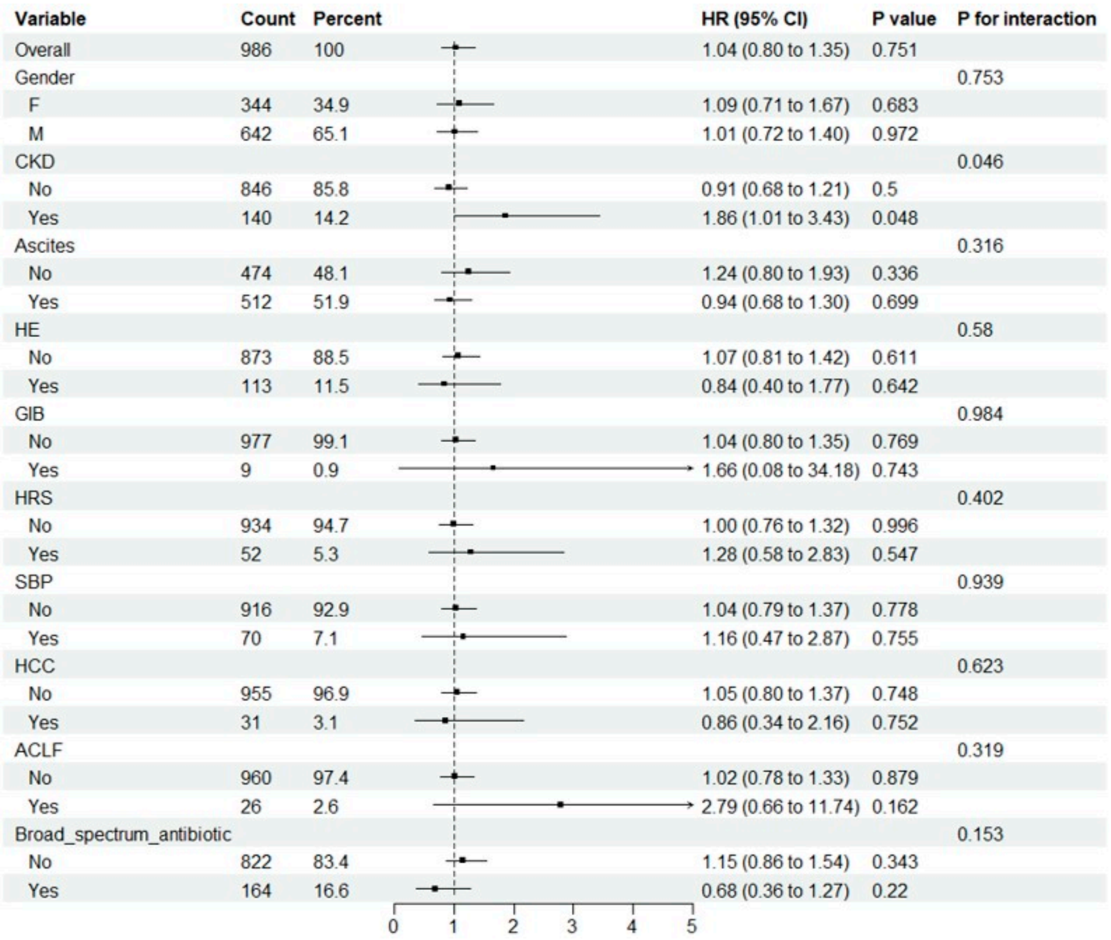

Supplementary Figure S8. Subgroup analysis of the effect of rifaximin on the risk of 180-day death. F, female; M, male; CKD, chronic kidney disease; HE, hepatic encephalopathy; SBP, spontaneous bacterial peritonitis; HRS, hepatorenal syndrome; ACLF, acute-on-chronic liver failure; GIB, gastrointestinal bleeding; HCC, hepatocellular carcinoma.

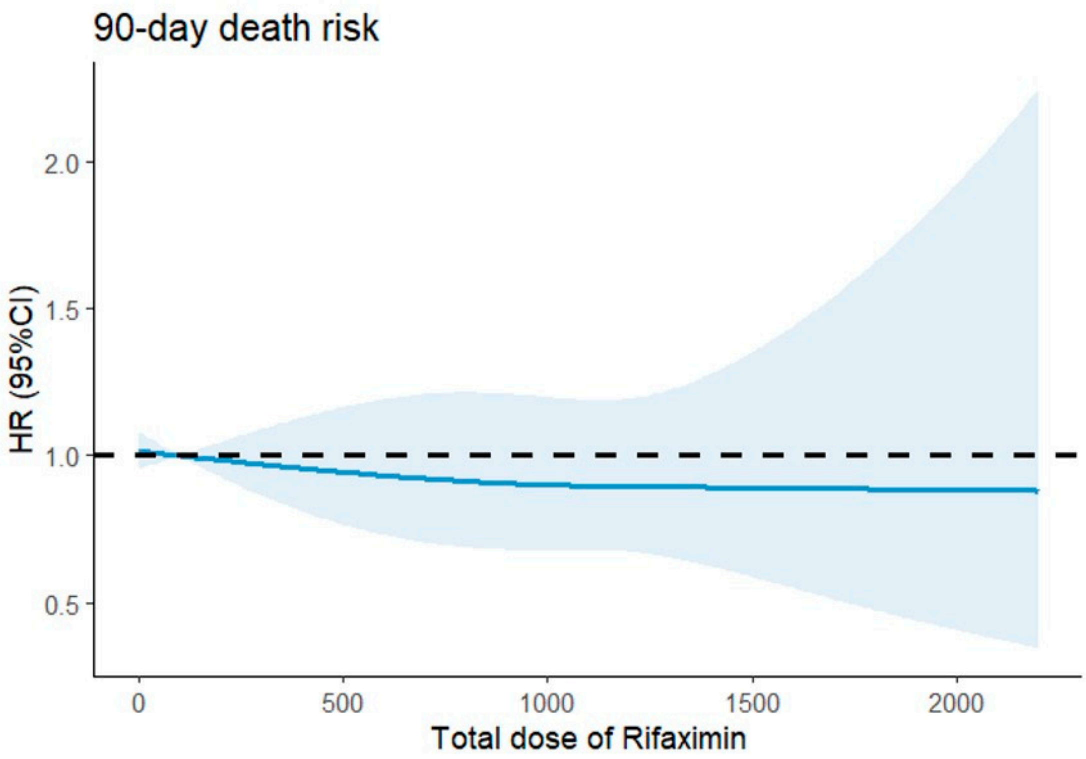

Supplementary Figure S9. Restricted cubic spline regression analysis the relationship between total dose of rifaximin and the risk of 90-day death. HR, hazard ratios; CI, confidence intervals.

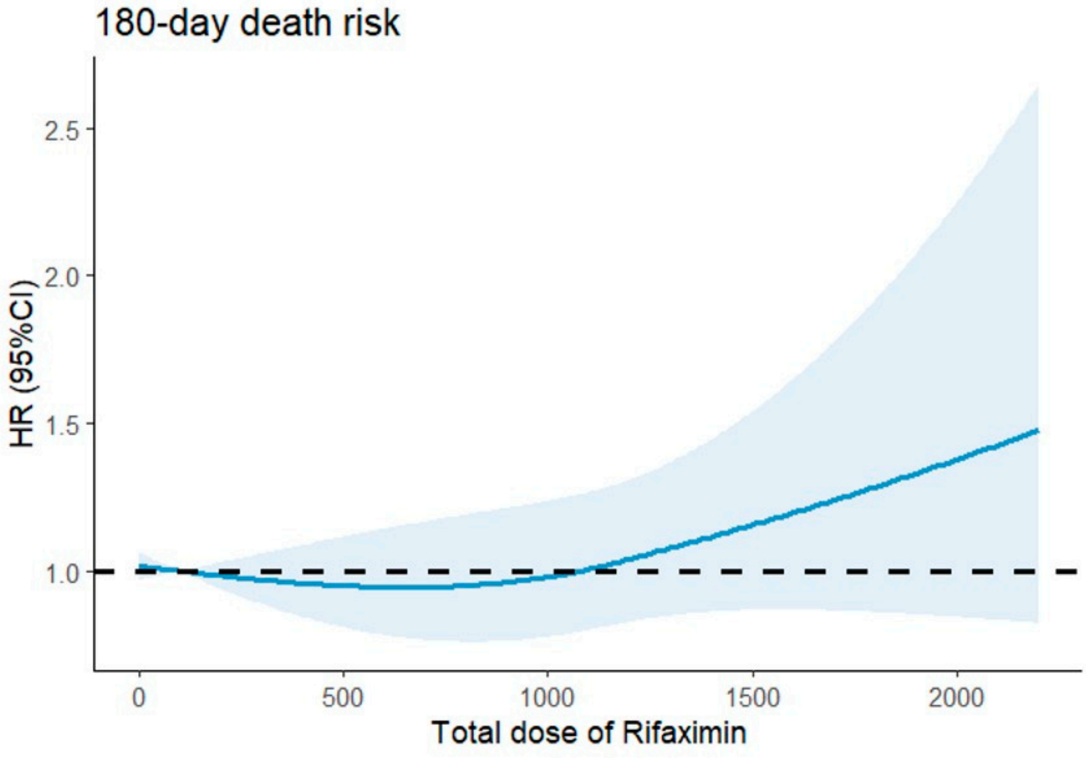

Supplementary Figure S10. Restricted cubic spline regression analysis the relationship between total dose of rifaximin and the risk of 180-day death. HR, hazard ratios; CI, confidence intervals.

Supplementary Table S1: Liver cirrhosis and its complications and comorbidity codes from ICD-9-CM to ICD-10-CM.

Category	ICD-9-CM codes	ICD-10-codes
**Liver cirrhosis**	5712, 5715, 5716	K703, K7030, K7031, K717, K74, K743, K744, K745, K746, K7460, K7469, P7881
**Hepatocellular carcinoma**	2308	C220,C227
**Spontaneous bacterial peritonitis**	56723	K652
**Ascites**	78951, 78959	K7011, K7031, K7151, R18
**Hepatic encephalopathy**	5722	K7291
**Gastrointestinal bleeding**	53082,5310,5312,5314,5316,5320,5322,5324,5326,5330,5332,5334,5336,5340,5342,5344,5346,5350,5351,5352,5353,53541,	K226,K250,K252,K254,K256,K260,K262,K264,K266,K270,K272,K274,K276,K280,K282,K284,K286
**Hepatorenal syndrome**	5724	K767
**Acute-on-chronic liver failure**	\	K720, K7200, K7201
**Other liver failure**	\	K704, K72, K721, K7210, K729, K7290, K7291
**Viral hepatitis**	070	B15,B16,B17,B18,B19
**Alcohol hepatitis**	5711	K70
**Autoimmune hepatitis**	57142	K754
**Non-alcoholic fatty liver disease**	5718	K7581
**Chronic kidney disease**	585	N18
**Diabetes**	250	E08,E10,E11,E13
**Hypertension**	401	I10

Supplementary Table S2. Protocol-defined broad-spectrum antibiotic regimens

Monotherapy	Gram-positive/negative	Gram-positive	Gram-negative	Anaerobic
Ampicillin-sulbactam	Cefazolin	Vancomycin	Aminoglycosides	Metronidazole
Amoxicillin-clavulanate	Cephalexin	Linezolid	Polymyxin B and colistin	Clindamycin
Piperacillin-tazobactam	Cefuroxime	Daptomycin	Aztreonam	
Cefoxitin	Cefdinir	Quinupristin-dalfopristin	Cefiderocol	
Meropenem +/- vaborbactam	Cefixime	Rifampin		
Imipinem-cilastatin +/- relebactam	Ceftazidime +/- avibactam	Rifabutin		
Ertapenem	Ceftriaxone	Penicillins		
Tigecycline	Ceftaroline			
Eravacycline	Ceftolozane-tazobactam			
	Ciprofloxacin			
	Levofloxacin			
	Doxycycline			
	Sulfamethoxazole-trimethoprim			
	Cefepime			

Note: Broad-spectrum antibiotic therapy is defined as: (1) Any agent from “Monotherapy”; (2) one agent from each of “Gram-positive/negative” and “Anaerobic”; (3) one agent from each of “Gram-positive,” “Gram-negative,” and “Anaerobic.”

Supplementary Table S3. Baseline characteristics of prevention cohort before PSM

Variables	Overall (n=4917)	No-rifaximin (n=4114)	Rifaximin (n=803)	P Value
**Age (median [IQR])**	59.66 [52.33, 68.06]	60.16 [52.56, 68.89]	57.79 [51.41, 64.97]	<0.001
**Gender (female) (%)**	1740 (35.4)	1453 (35.3)	287 (35.7)	0.85
**Race (%)**				
Asian	170 (3.5)	153 (3.7)	17 (2.1)	<0.001
Black	430 (8.7)	389 (9.5)	41 (5.1)	
White	3456 (70.3)	2879 (70.0)	577 (71.9)	
Other	861 (17.5)	693 (16.8)	168 (20.9)	
**Admission type (%)**				<0.001
Elective	77 (1.6)	74 (1.8)	3 (0.4)	
Observation	887 (18.0)	710 (17.3)	177 (22.0)	
Surgical	307 (6.2)	300 (7.3)	7 (0.9)	
Urgent	3646 (74.2)	3030 (73.7)	616 (76.7)	
**Hypertension (%)**	1775 (36.1)	1547 (37.6)	228 (28.4)	<0.001
**Diabetes (%)**	1525 (31.0)	1281 (31.1)	244 (30.4)	0.704
**CKD (%)**	794 (16.1)	668 (16.2)	126 (15.7)	0.74
**Infection (%)**	3253 (66.2)	2702 (65.7)	551 (68.6)	0.117
**Etiology of liver cirrhosis**				
Viral hepatitis (%)	1650 (33.6)	1370 (33.3)	280 (34.9)	0.412
Alcohol hepatitis (%)	1200 (24.4)	927 (22.5)	273 (34.0)	<0.001
Autoimmune hepatitis (%)	97 (2.0)	79 (1.9)	18 (2.2)	0.645
NAFLD (%)	265 (5.4)	209 (5.1)	56 (7.0)	0.037
**Ascites (%)**	1689 (34.4)	1206 (29.3)	483 (60.1)	<0.001
**HE (%)**	315 (6.4)	154 (3.7)	161 (20.0)	<0.001
**GIB (%)**	113 (2.3)	97 (2.4)	16 (2.0)	0.615
**HRS (%)**	257 (5.2)	141 (3.4)	116 (14.4)	<0.001
**HCC (%)**	126 (2.6)	101 (2.5)	25 (3.1)	0.338
**ACLF (%)**	118 (2.4)	79 (1.9)	39 (4.9)	<0.001
**SBP (%)**	234 (4.8)	158 (3.8)	76 (9.5)	<0.001
**Other liver failure (%)**	499 (10.1)	274 (6.7)	225 (28.0)	<0.001
**Hb (g/dL) (median [IQR])**	10.70 [9.20, 12.30]	10.90 [9.30, 12.40]	10.20 [8.80, 11.50]	<0.001
**WBC (10^9/L) (median [IQR])**	6.90 [4.70, 10.10]	7.00 [4.80, 10.20]	6.30 [4.20, 9.60]	<0.001
**PLT (10^9/L) (median [IQR])**	120.00 [76.00, 182.00]	126.00 [80.00, 190.00]	89.00 [61.00, 136.00]	<0.001
**TBIL (mg/dL) (median [IQR])**	1.60 [0.80, 3.90]	1.40 [0.70, 3.40]	3.20 [1.60, 7.80]	<0.001
**ALT (U/L) (median [IQR])**	34.00 [21.00, 62.00]	34.00 [21.00, 64.00]	35.00 [22.00, 55.00]	0.903
**AST (U/L) (median [IQR])**	60.00 [36.00, 114.00]	59.00 [34.00, 114.00]	69.00 [42.00, 113.50]	<0.001
**ALB (g/dL) (median [IQR])**	3.10 [2.70, 3.60]	3.10 [2.70, 3.60]	2.90 [2.50, 3.40]	<0.001
**ALP (U/L) (median [IQR])**	113.00 [81.00, 169.00]	111.00 [79.00, 168.00]	125.00 [91.50, 175.00]	<0.001

**Scr (mg/dL) (median [IQR])**	0.90 [0.70, 1.30]	0.90 [0.70, 1.30]	1.00 [0.70, 1.60]	<0.001
**PT (s) (median [IQR])**	15.50 [13.40, 19.10]	15.00 [13.10, 18.40]	18.10 [15.30, 22.40]	<0.001
**INR (median [IQR])**	1.40 [1.20, 1.70]	1.40 [1.20, 1.70]	1.70 [1.40, 2.10]	<0.001
**Na (mmol/L) (median [IQR])**	137.00 [134.00, 140.00]	137.00 [134.00, 140.00]	136.00 [131.00, 139.00]	<0.001
**K (mmol/L) (median [IQR])**	4.00 [3.60, 4.40]	4.00 [3.60, 4.40]	4.00 [3.60, 4.40]	0.771
**MELD (median [IQR])**	14.58 [10.20, 20.59]	13.63 [10.00, 19.51]	19.04 [14.17, 25.21]	<0.001
**Ventilation (%)**	323 (6.6)	272 (6.6)	51 (6.4)	0.846
**Liver transplantation (%)**	25 (0.5)	21 (0.5)	4 (0.5)	1
**Hemodialysis (%)**	154 (3.1)	121 (2.9)	33 (4.1)	0.104
**Total dose of rifaximin (mg) (median [IQR])**	\	\	1100.00 [1100.00, 1100.00]	\
**Broad spectrum antibiotic (%)**	709 (14.4)	570 (13.9)	139 (17.3)	0.013
**Human albumin (%)**	1112 (22.6)	657 (16.0)	455 (56.7)	<0.001
**Vasoactive agent (%)**	28 (0.6)	16 (0.4)	12 (1.5)	<0.001

Abbreviations: ICU, intensive care unit; IQR, interquartile range; HE, hepatic encephalopathy; SBP, spontaneous bacterial peritonitis; HRS, hepatorenal syndrome; ACLF, acute-on-chronic liver failure; NALFD, non-alcoholic fatty liver disease; GIB, gastrointestinal bleeding; HCC, hepatocellular carcinoma; Hb, hemoglobin; WBC, white blood cell; PLT, platelet, TBIL, total bilirubin; ALT, alanine aminotransferase; AST, aspartate aminotransferase; ALB, serum albumin; ALP, alkaline phosphatase; Scr, serum creatinine; Na, sodium; K, potassium; PT, prothrombin time; INR, international normalized ratio; MELD, model for end-stage liver disease.

Supplementary Table S4. Baseline characteristics of prevention cohort after PSM

Variables	Overall (n=1326)	No rifaximin (n=663)	Rifaximin (n=663)	P Value
**Age (median [IQR])**	57.78 [51.26, 65.85]	57.45 [50.36, 66.83]	58.05 [51.70, 65.18]	0.996
**Gender (%)**	477 (36.0)	242 (36.5)	235 (35.4)	0.731
**Race (%)**				0.183
Asian	22 (1.7)	6 (0.9)	16 (2.4)	
Black	79 (6.0)	41 (6.2)	38 (5.7)	
White	973 (73.4)	492 (74.2)	481 (72.5)	
Other	252 (19.0)	124 (18.7)	128 (19.3)	
**Admission type (%)**				
Elective	9 (0.7)	6 (0.9)	3 (0.5)	0.768
Observation	278 (21.0)	136 (20.5)	142 (21.4)	
Surgical	14 (1.1)	7 (1.1)	7 (1.1)	
Urgent	1025 (77.3)	514 (77.5)	511 (77.1)	
**Hypertension (%)**	359 (27.1)	164 (24.7)	195 (29.4)	0.064
**Diabetes (%)**	390 (29.4)	189 (28.5)	201 (30.3)	0.507
**CKD (%)**	214 (16.1)	111 (16.7)	103 (15.5)	0.601
**Infection (%)**	885 (66.7)	442 (66.7)	443 (66.8)	1
**Etiology of liver cirrhosis**				
Viral hepatitis (%)	497 (37.5)	251 (37.9)	246 (37.1)	0.82
Alcohol hepatitis (%)	424 (32.0)	211 (31.8)	213 (32.1)	0.953
Autoimmune hepatitis (%)	24 (1.8)	13 (2.0)	11 (1.7)	0.837
NAFLD (%)	87 (6.6)	42 (6.3)	45 (6.8)	0.824
**Ascites (%)**	757 (57.1)	386 (58.2)	371 (56.0)	0.437
**HE (%)**	192 (14.5)	92 (13.9)	100 (15.1)	0.585
**GIB (%)**	23 (1.7)	14 (2.1)	9 (1.4)	0.4
**HRS (%)**	137 (10.3)	66 (10.0)	71 (10.7)	0.718
**HCC (%)**	45 (3.4)	24 (3.6)	21 (3.2)	0.762
**ACLF (%)**	65 (4.9)	31 (4.7)	34 (5.1)	0.799
**SBP (%)**	112 (8.4)	54 (8.1)	58 (8.7)	0.767
**Other liver failure (%)**	314 (23.7)	159 (24.0)	155 (23.4)	0.846
**Hb (g/dL) (median [IQR])**	10.20 [8.80, 11.70]	10.20 [8.70, 11.60]	10.30 [8.90, 11.70]	0.573
**WBC (10^9/L) (median [IQR])**	6.20 [4.20, 9.40]	6.40 [4.30, 9.25]	6.10 [4.10, 9.45]	0.903
**PLT (10^9/L) (median [IQR])**	90.00 [61.00, 139.00]	91.00 [59.00, 139.00]	89.00 [62.00, 139.50]	0.775
**TBIL (mg/dL) (median [IQR])**	2.80 [1.40, 7.10]	2.80 [1.30, 7.60]	2.80 [1.50, 6.70]	0.471
**ALT (U/L) (median [IQR])**	34.00 [22.00, 55.00]	34.00 [22.00, 56.00]	34.00 [22.00, 54.00]	0.963
**AST (U/L) (median [IQR])**	69.00 [41.00, 117.00]	71.00 [41.00, 123.00]	67.00 [40.00, 112.00]	0.317
**ALB (g/dL) (median [IQR])**	2.90 [2.50, 3.40]	2.90 [2.50, 3.40]	2.90 [2.50, 3.40]	0.303
**ALP (U/L) (median [IQR])**	122.00 [88.25, 176.75]	117.00 [84.00, 177.50]	124.00 [92.00, 176.00]	0.063
**Scr (mg/dL) (median [IQR])**	0.90 [0.70, 1.50]	0.90 [0.70, 1.40]	1.00 [0.70, 1.50]	0.06

**PT (s) (median [IQR])**	17.60 [15.00, 21.60]	17.80 [14.80, 21.85]	17.50 [15.10, 21.40]	0.725
**INR (median [IQR])**	1.60 [1.40, 2.00]	1.60 [1.40, 2.00]	1.60 [1.40, 2.00]	0.73
**Na (mmol/L) (median [IQR])**	136.00 [132.00, 139.00]	136.00 [133.00, 139.00]	136.00 [132.00, 139.00]	0.584
**K (mmol/L) (median [IQR])**	4.00 [3.60, 4.40]	4.00 [3.60, 4.40]	4.00 [3.60, 4.40]	0.316
**MELD (median [IQR])**	18.14 [13.40, 24.20]	18.25 [13.40, 24.18]	18.01 [13.40, 24.24]	0.893
**Ventilation (%)**	75 (5.7)	46 (6.9)	29 (4.4)	0.057
**Liver transplantation (%)**	7 (0.5)	3 (0.5)	4 (0.6)	1
**Hemodialysis (%)**	45 (3.4)	21 (3.2)	24 (3.6)	0.762
**Total dose of rifaximin (mg) (median [IQR])**	\	\	1100.00 [1100.00, 1100.00]	\
**Broad spectrum antibiotic (%)**	205 (15.5)	98 (14.8)	107 (16.1)	0.543
**Human albumin (%)**	611 (46.1)	292 (44.0)	319 (48.1)	0.152
**Vasoactive agent (%)**	11 (0.8)	7 (1.1)	4 (0.6)	0.545

Abbreviations: ICU, intensive care unit; IQR, interquartile range; HE, hepatic encephalopathy; SBP, spontaneous bacterial peritonitis; HRS, hepatorenal syndrome; ACLF, acute-on-chronic liver failure; NALFD, non-alcoholic fatty liver disease; GIB, gastrointestinal bleeding; HCC, hepatocellular carcinoma; Hb, hemoglobin; WBC, white blood cell; PLT, platelet, TBIL, total bilirubin; ALT, alanine aminotransferase; AST, aspartate aminotransferase; ALB, serum albumin; ALP, alkaline phosphatase; Scr, serum creatinine; Na, sodium; K, potassium; PT, prothrombin time; INR, international normalized ratio; MELD, model for end-stage liver disease.

Supplementary Table S5. Baseline characteristics of ICU sub-cohort before PSM

Variables	Overall (n=1910)	No rifaximin (n=1477)	Rifaximin (n=433)	P Value
**Age (median [IQR])**	59.78 [52.25, 68.59]	60.97 [52.95, 69.87]	56.99 [49.52, 64.10]	<0.001
**Gender (%)**	640 (33.5)	481 (32.6)	159 (36.7)	0.12
**Race (%)**				0.01
Asian	39 (2.0)	36 (2.4)	3 (0.7)	
Black	120 (6.3)	98 (6.6)	22 (5.1)	
White	1220 (63.9)	953 (64.5)	267 (61.7)	
Other	531 (27.8)	390 (26.4)	141 (32.6)	
**Admission type (%)**				<0.001
Elective	22 (1.2)	21 (1.4)	1 (0.2)	
Observation	226 (11.8)	189 (12.8)	37 (8.5)	
Surgical	75 (3.9)	70 (4.7)	5 (1.2)	
Urgent	1587 (83.1)	1197 (81.0)	390 (90.1)	
**SOFA (median [IQR])**	7.00 [4.00, 10.00]	6.00 [4.00, 9.00]	9.00 [7.00, 12.00]	<0.001
**MELD (median [IQR])**	19.16 [13.40, 28.08]	17.44 [12.38, 25.55]	27.04 [18.94, 33.97]	<0.001
**Hypertension (%)**	663 (34.7)	533 (36.1)	130 (30.0)	0.023
**Diabetes (%)**	564 (29.5)	456 (30.9)	108 (24.9)	0.02
**CKD (%)**	356 (18.6)	284 (19.2)	72 (16.6)	0.25
**Infection (%)**	1690 (88.5)	1286 (87.1)	404 (93.3)	<0.001
**Etiology of liver cirrhosis (%)**				
Viral hepatitis	528 (27.6)	400 (27.1)	128 (29.6)	0.34
Alcohol hepatitis	595 (31.2)	403 (27.3)	192 (44.3)	<0.001
Autoimmune hepatitis	32 (1.7)	24 (1.6)	8 (1.8)	0.917
NAFLD	79 (4.1)	65 (4.4)	14 (3.2)	0.349
**Ascites (%)**	802 (42.0)	533 (36.1)	269 (62.1)	<0.001
**HE (%)**	201 (10.5)	101 (6.8)	100 (23.1)	<0.001
**GIB (%)**	98 (5.1)	75 (5.1)	23 (5.3)	0.944
**HRS (%)**	206 (10.8)	114 (7.7)	92 (21.2)	<0.001
**HCC (%)**	37 (1.9)	29 (2.0)	8 (1.8)	1
**ACLF (%)**	108 (5.7)	60 (4.1)	48 (11.1)	<0.001
**SBP (%)**	146 (7.6)	93 (6.3)	53 (12.2)	<0.001
**Other liver failure (%)**	339 (17.7)	180 (12.2)	159 (36.7)	<0.001
**Hb (g/dL) (median [IQR])**	10.30 [9.00, 11.90]	10.40 [9.10, 12.00]	10.00 [8.60, 11.30]	<0.001
**WBC (10^9/L) (median [IQR])**	10.90 [7.20, 16.40]	10.60 [7.20, 16.00]	11.60 [7.40, 17.70]	0.013
**PLT (10^9/L) (median [IQR])**	127.00 [82.00, 192.00]	134.00 [88.00, 202.00]	103.00 [71.00, 154.00]	<0.001
**TBIL (mg/dL) (median [IQR])**	2.40 [1.10, 6.20]	1.90 [0.90, 4.50]	5.30 [2.30, 13.20]	<0.001
**ALT (U/L) (median [IQR])**	35.00 [21.00, 70.00]	34.00 [20.00, 65.00]	40.00 [23.00, 83.00]	<0.001
**AST (U/L) (median [IQR])**	69.00 [38.00, 146.00]	63.00 [36.00, 136.00]	94.00 [48.00, 194.00]	<0.001
**ALB (g/dL) (median [IQR])**	3.10 [2.60, 3.50]	3.10 [2.70, 3.50]	2.90 [2.50, 3.50]	<0.001

**ALP (U/L) (median [IQR])**	107.50 [75.00, 163.00]	105.00 [74.00, 160.00]	116.00 [84.00, 173.00]	0.001
**Scr (mg/dL) (median [IQR])**	1.20 [0.80, 2.20]	1.20 [0.80, 2.00]	1.70 [1.00, 2.90]	<0.001
**PT (s) (median [IQR])**	17.90 [14.70, 23.98]	17.10 [14.30, 22.30]	21.70 [17.50, 28.50]	<0.001
**INR (median [IQR])**	1.60 [1.30, 2.20]	1.60 [1.30, 2.10]	2.00 [1.60, 2.70]	<0.001
**Na (mmol/L) (median [IQR])**	139.00 [136.00, 142.00]	139.00 [136.00, 142.00]	139.00 [135.00, 143.00]	0.455
**K (mmol/L) (median [IQR])**	4.40 [4.00, 5.00]	4.40 [4.00, 5.00]	4.40 [4.00, 5.20]	0.959
**Ventilation (%)**	427 (22.4)	297 (20.1)	130 (30.0)	<0.001
**Liver transplantation (%)**	26 (1.4)	23 (1.6)	3 (0.7)	0.259
**Hemodialysis (%)**	142 (7.4)	95 (6.4)	47 (10.9)	0.003
**Total dose of rifaximin (mg) (median [IQR])**	0.00 [0.00, 0.00]	0.00 [0.00, 0.00]	1100.00 [1100.00, 1200.00]	<0.001
**Pre-rifaximin (%)**	677 (35.4)	244 (16.5)	433 (100.0)	<0.001
**Post-rifaximin (%)**	533 (27.9)	209 (14.2)	324 (74.8)	<0.001
**Broad spectrum antibiotic (%)**	592 (31.0)	407 (27.6)	185 (42.7)	<0.001
**Human albumin (%)**	744 (39.0)	473 (32.0)	271 (62.6)	<0.001
**Vasoactive agent (%)**	633 (33.1)	431 (29.2)	202 (46.7)	<0.001
**Heart rate (median [IQR])**	103.00 [90.00, 119.00]	103.00 [90.00, 119.00]	103.00 [90.00, 122.00]	0.544
**RR (median [IQR])**	27.00 [23.00, 32.00]	27.00 [23.00, 31.00]	27.00 [24.00, 32.00]	0.177
**Spo2 (median [IQR])**	100.00 [99.00, 100.00]	100.00 [99.00, 100.00]	100.00 [99.00, 100.00]	0.575
**Anion gap (median [IQR])**	17.00 [13.00, 20.75]	16.00 [13.00, 20.00]	17.00 [14.00, 22.00]	<0.001
**Sbpressure (median [IQR])**	140.00 [127.00, 156.00]	141.00 [128.00, 157.00]	136.00 [124.00, 152.00]	<0.001
**Dbpressure (median [IQR])**	84.00 [74.00, 97.00]	85.00 [74.00, 98.00]	82.00 [71.00, 95.00]	0.011
**Temperature (median [IQR])**	37.17 [36.89, 37.56]	37.17 [36.89, 37.60]	37.11 [36.89, 37.50]	0.075

Abbreviations: ICU, intensive care unit; IQR, interquartile range; HE, hepatic encephalopathy; SBP, spontaneous bacterial peritonitis; HRS, hepatorenal syndrome; ACLF, acute-on-chronic liver failure; NALFD, non-alcoholic fatty liver disease; GIB, gastrointestinal bleeding; HCC, hepatocellular carcinoma; Hb, hemoglobin; WBC, white blood cell; PLT, platelet, TBIL, total bilirubin; ALT, alanine aminotransferase; AST, aspartate aminotransferase; ALB, serum albumin; ALP, alkaline phosphatase; Scr, serum creatinine; Na, sodium; K, potassium; PT, prothrombin time; INR, international normalized ratio; MELD, model for end-stage liver disease. RR, respiratory rate; SPO2, pulse oxygen saturation; SOFA, sequential organ failure assessment; Dbpressure, diastolic blood pressure; Sbpressure, systolic blood pressure.

Supplementary Table S6. Baseline characteristics of ICU sub-cohort after PSM

Variables	Overall (n=456)	No (n=228)	Yes (n=228)	P Value
**Age (median [IQR])**	57.03 [50.78, 64.18]	56.91 [50.95, 64.60]	57.12 [50.30, 63.95]	0.733
**Gender (%)**	184 (40.4)	87 (38.2)	97 (42.5)	0.39
**Race (%)**				0.912
Asian	3 (0.7)	2 (0.9)	1 (0.4)	
Black	24 (5.3)	11 (4.8)	13 (5.7)	
White	287 (62.9)	143 (62.7)	144 (63.2)	
Other	142 (31.1)	72 (31.6)	70 (30.7)	
**Admission type (%)**				0.851
Elective	0	0	0	
Observation	53 (11.6)	28 (12.3)	25 (11.0)	
Surgical	7 (1.5)	3 (1.3)	4 (1.8)	
Urgent	396 (86.8)	197 (86.4)	199 (87.3)	
**SOFA score (median [IQR])**	9.00 [6.00, 12.00]	9.00 [6.00, 12.00]	9.00 [7.00, 11.25]	0.883
**MELD score (median [IQR])**	25.84 [18.24, 34.74]	25.59 [18.13, 35.14]	25.92 [18.58, 34.45]	0.958
**Hypertension (%)**	132 (28.9)	64 (28.1)	68 (29.8)	0.757
**Diabetes (%)**	111 (24.3)	59 (25.9)	52 (22.8)	0.513
**CKD (%)**	90 (19.7)	52 (22.8)	38 (16.7)	0.126
**Infection (%)**	440 (96.5)	221 (96.9)	219 (96.1)	0.799
**Etiology of liver cirrhosis**				
Viral hepatitis (%)	136 (29.8)	68 (29.8)	68 (29.8)	1
Alcohol hepatitis (%)	201 (44.1)	103 (45.2)	98 (43.0)	0.706
Autoimmune hepatitis (%)	10 (2.2)	5 (2.2)	5 (2.2)	1
NAFLD (%)	16 (3.5)	9 (3.9)	7 (3.1)	0.799
**Ascites (%)**	305 (66.9)	162 (71.1)	143 (62.7)	0.073
**HE (%)**	107 (23.5)	55 (24.1)	52 (22.8)	0.825
**GIB (%)**	30 (6.6)	16 (7.0)	14 (6.1)	0.85
**HRS (%)**	101 (22.1)	57 (25.0)	44 (19.3)	0.176
**HCC (%)**	11 (2.4)	5 (2.2)	6 (2.6)	1
**ACLF (%)**	48 (10.5)	21 (9.2)	27 (11.8)	0.445
**SBP (%)**	69 (15.1)	37 (16.2)	32 (14.0)	0.601
**Other liver failure (%)**	163 (35.7)	82 (36.0)	81 (35.5)	1
**Hb (g/dL) (median [IQR])**	9.60 [8.40, 10.72]	9.60 [8.47, 10.60]	9.60 [8.40, 11.00]	0.364
**WBC (10^9/L) (median [IQR])**	10.55 [7.30, 16.70]	10.30 [7.50, 16.47]	10.90 [7.27, 16.88]	0.85
**PLT (10^9/L) (median [IQR])**	103.00 [71.00, 155.25]	106.00 [71.75, 156.00]	98.50 [70.00, 155.00]	0.565
**TBIL (mg/dL) (median [IQR])**	5.10 [2.30, 13.67]	4.80 [2.30, 14.33]	5.25 [2.20, 12.90]	0.949
**ALT (U/L) (median [IQR])**	36.00 [22.00, 69.00]	35.00 [22.00, 64.00]	37.00 [22.75, 72.75]	0.43
**AST (U/L) (median [IQR])**	82.00 [44.00, 153.00]	71.50 [41.75, 143.75]	89.00 [48.75, 162.50]	0.186
**ALB (g/dL) (median [IQR])**	3.10 [2.60, 3.60]	3.20 [2.60, 3.60]	3.00 [2.50, 3.60]	0.128

**ALP (U/L) (median [IQR])**	109.50 [77.00, 162.00]	116.00 [76.75, 165.00]	107.50 [77.00, 153.00]	0.484
**Scr (mg/dL) (median [IQR])**	1.55 [0.90, 2.80]	1.55 [0.90, 3.00]	1.55 [0.90, 2.70]	0.652
**PT (s) (median [IQR])**	21.70 [16.98, 28.02]	22.00 [17.00, 27.70]	21.20 [16.90, 28.15]	0.995
**INR (median [IQR])**	2.00 [1.58, 2.60]	2.05 [1.60, 2.50]	2.00 [1.50, 2.70]	0.896
**Na (mmol/L) (median [IQR])**	139.00 [135.00, 143.00]	139.00 [135.00, 143.00]	139.00 [135.00, 142.00]	0.914
**K (mmol/L) (median [IQR])**	4.30 [3.90, 5.10]	4.30 [3.90, 5.10]	4.40 [3.90, 5.10]	0.937
**Ventilation (%)**	114 (25.0)	54 (23.7)	60 (26.3)	0.589
**Liver transplantation (%)**	6 (1.3)	4 (1.8)	2 (0.9)	0.681
**Hemodialysis (%)**	46 (10.1)	24 (10.5)	22 (9.6)	0.876
**Total dose of rifaximin (mg) (median [IQR])**	\	\	1100.00 [1100.00, 1200.00]	
**Pre-rifaximin (%)**	456 (100.0)	228 (100.0)	228 (100.0)	NA
**Post-rifaximin (%)**	385 (84.4)	194 (85.1)	191 (83.8)	0.796
**Broad spectrum antibiotic (%)**	159 (34.9)	74 (32.5)	85 (37.3)	0.326
**Human albumin (%)**	228 (50.0)	111 (48.7)	117 (51.3)	0.64
**Vasoactive agent (%)**	168 (36.8)	82 (36.0)	86 (37.7)	0.771
**Heart rate (median [IQR])**	102.00 [89.00, 116.25]	103.00 [90.00, 115.00]	100.00 [87.00, 117.25]	0.358
**RR (median [IQR])**	27.00 [23.00, 32.00]	27.00 [23.00, 32.00]	27.00 [23.88, 32.00]	0.825
**Spo2 (median [IQR])**	100.00 [99.00, 100.00]	100.00 [99.00, 100.00]	100.00 [99.00, 100.00]	0.721
**Anion gap (median [IQR])**	17.00 [14.00, 21.25]	17.00 [14.00, 22.00]	17.00 [14.00, 21.00]	0.973
**Sbpressure (median [IQR])**	136.00 [124.00, 153.00]	137.50 [123.38, 157.25]	135.00 [124.00, 149.00]	0.172
**Dbpressure (median [IQR])**	83.00 [71.00, 98.00]	84.00 [71.75, 99.00]	82.00 [71.00, 94.25]	0.224
**Temperature (median [IQR])**	37.06 [36.89, 37.50]	37.06 [36.83, 37.50]	37.11 [36.89, 37.44]	0.333

Abbreviations: ICU, intensive care unit; IQR, interquartile range; HE, hepatic encephalopathy; SBP, spontaneous bacterial peritonitis; HRS, hepatorenal syndrome; ACLF, acute-on-chronic liver failure; NALFD, non-alcoholic fatty liver disease; GIB, gastrointestinal bleeding; HCC, hepatocellular carcinoma; Hb, hemoglobin; WBC, white blood cell; PLT, platelet, TBIL, total bilirubin; ALT, alanine aminotransferase; AST, aspartate aminotransferase; ALB, serum albumin; ALP, alkaline phosphatase; Scr, serum creatinine; Na, sodium; K, potassium; PT, prothrombin time; INR, international normalized ratio; MELD, model for end-stage liver disease; RR, respiratory rate; SPO2, pulse oxygen saturation; SOFA, sequential organ failure assessment; Dbpressure, diastolic blood pressure; Sbpressure, systolic blood pressure.

Supplementary Table S7. Baseline characteristics of non-ICU sub-cohort before PSM

Variables	Overall (n=3471)	No rifaximin (n=2811)	Rifaximin (n=660)	P Value
**Age (median [IQR])**	59.65 [52.29, 67.82]	60.18 [52.57, 68.64]	57.84 [51.43, 65.14]	<0.001
**Gender (%)**	1254 (36.1)	1023 (36.4)	231 (35.0)	0.532
**Race (%)**				<0.001
Asian	134 (3.9)	117 (4.2)	17 (2.6)	
Black	333 (9.6)	300 (10.7)	33 (5.0)	
White	2510 (72.3)	2018 (71.8)	492 (74.5)	
Other	494 (14.2)	376 (13.4)	118 (17.9)	
**Admission type (%)**				<0.001
Elective	56 (1.6)	53 (1.9)	3 (0.5)	
Observation	690 (19.9)	533 (19.0)	157 (23.8)	
Surgical	232 (6.7)	226 (8.0)	6 (0.9)	
Urgent	2493 (71.8)	1999 (71.1)	494 (74.8)	
**Hypertension (%)**	1282 (36.9)	1088 (38.7)	194 (29.4)	<0.001
**Diabetes (%)**	1101 (31.7)	898 (31.9)	203 (30.8)	0.587
**CKD (%)**	523 (15.1)	433 (15.4)	90 (13.6)	0.279
**Infection (%)**	1975 (56.9)	1563 (55.6)	412 (62.4)	0.002
**Etiology of liver cirrhosis**				
Viral hepatitis (%)	1239 (35.7)	997 (35.5)	242 (36.7)	0.594
Alcohol hepatitis (%)	741 (21.3)	534 (19.0)	207 (31.4)	<0.001
Autoimmune hepatitis (%)	74 (2.1)	60 (2.1)	14 (2.1)	1
NAFLD (%)	205 (5.9)	156 (5.5)	49 (7.4)	0.081
**Ascites (%)**	1089 (31.4)	721 (25.6)	368 (55.8)	<0.001
**HE (%)**	177 (5.1)	52 (1.8)	125 (18.9)	<0.001
**GIB (%)**	41 (1.2)	30 (1.1)	11 (1.7)	0.279
**HRS (%)**	96 (2.8)	37 (1.3)	59 (8.9)	<0.001
**HCC (%)**	93 (2.7)	72 (2.6)	21 (3.2)	0.451
**ACLF (%)**	38 (1.1)	14 (0.5)	24 (3.6)	<0.001
**SBP (%)**	121 (3.5)	73 (2.6)	48 (7.3)	<0.001
**Other liver failure (%)**	239 (6.9)	70 (2.5)	169 (25.6)	<0.001
**Hb (g/dL) (median [IQR])**	11.00 [9.50, 12.40]	11.10 [9.60, 12.60]	10.30 [8.90, 11.70]	<0.001
**WBC (10^9/L) (median [IQR])**	6.50 [4.40, 9.10]	6.50 [4.50, 9.20]	5.80 [4.07, 8.80]	0.001
**PLT (10^9/L) (median [IQR])**	121.00 [76.00, 182.50]	130.00 [83.00, 192.00]	88.00 [61.00, 136.00]	<0.001
**TBIL (mg/dL) (median [IQR])**	1.50 [0.80, 3.40]	1.30 [0.70, 2.80]	2.80 [1.50, 6.03]	<0.001
**ALT (U/L) (median [IQR])**	34.00 [21.00, 61.00]	34.00 [21.00, 64.00]	34.50 [22.00, 53.00]	0.885
**AST (U/L) (median [IQR])**	59.00 [35.00, 105.00]	57.00 [34.00, 104.00]	65.00 [41.00, 110.25]	<0.001
**ALB (g/dL) (median [IQR])**	3.20 [2.70, 3.60]	3.20 [2.80, 3.70]	2.90 [2.50, 3.40]	<0.001
**ALP (U/L) (median [IQR])**	116.00 [83.00, 169.00]	112.00 [80.00, 167.00]	127.00 [94.00, 176.25]	<0.001
**Scr (mg/dL) (median [IQR])**	0.90 [0.70, 1.20]	0.90 [0.70, 1.20]	0.90 [0.70, 1.40]	<0.001

**PT (s) (median [IQR])**	15.10 [13.20, 18.30]	14.60 [13.00, 17.40]	17.50 [15.00, 21.22]	<0.001
**INR (median [IQR])**	1.40 [1.20, 1.70]	1.30 [1.20, 1.60]	1.60 [1.40, 2.00]	<0.001
**Na (mmol/L) (median [IQR])**	137.00 [134.00, 140.00]	138.00 [135.00, 140.00]	136.00 [132.00, 139.00]	<0.001
**K (mmol/L) (median [IQR])**	4.00 [3.60, 4.30]	4.00 [3.60, 4.30]	4.00 [3.60, 4.32]	0.928
**MELD (median [IQR])**	13.62 [10.00, 19.02]	12.62 [9.37, 17.85]	17.75 [13.46, 23.48]	<0.001
**Ventilation (%)**	43 (1.2)	24 (0.9)	19 (2.9)	<0.001
**Liver transplantation (%)**	0 (0)	0 (0)	0 (0)	\
**Hemodialysis (%)**	52 (1.5)	39 (1.4)	13 (2.0)	0.352
**Total dose of rifaximin (mg) (median [IQR])**	\	\	1100.00 [1100.00, 1100.00]	\
**Broad spectrum antibiotic (%)**	551 (15.9)	449 (16.0)	102 (15.5)	0.788
**Human albumin (%)**	882 (25.4)	543 (19.3)	339 (51.4)	<0.001
**Vasoactive agent (%)**	23 (0.7)	13 (0.5)	10 (1.5)	0.006

Abbreviations: ICU, intensive care unit; IQR, interquartile range; HE, hepatic encephalopathy; SBP, spontaneous bacterial peritonitis; HRS, hepatorenal syndrome; ACLF, acute-on-chronic liver failure; NALFD, non-alcoholic fatty liver disease; GIB, gastrointestinal bleeding; HCC, hepatocellular carcinoma; Hb, hemoglobin; WBC, white blood cell; PLT, platelet, TBIL, total bilirubin; ALT, alanine aminotransferase; AST, aspartate aminotransferase; ALB, serum albumin; ALP, alkaline phosphatase; Scr, serum creatinine; Na, sodium; K, potassium; PT, prothrombin time; INR, international normalized ratio; MELD, model for end-stage liver disease.

Supplementary Table S8. Baseline characteristics of non-ICU sub-cohort after PSM

Variables	Overall (n=986)	No rifaximin (n=493)	Rifaximin (n=493)	P Value
**Age (median [IQR])**	57.98 [51.46, 65.42]	57.84 [51.04, 65.54]	58.03 [51.69, 65.30]	0.934
**Gender (%)**	344 (34.9)	168 (34.1)	176 (35.7)	0.64
**Race (%)**				0.862
Asian	25 (2.5)	11 (2.2)	14 (2.8)	
Black	57 (5.8)	28 (5.7)	29 (5.9)	
White	752 (76.3)	381 (77.3)	371 (75.3)	
Other	152 (15.4)	73 (14.8)	79 (16.0)	
**Admission type (%)**				0.976
Elective	6 (0.6)	3 (0.6)	3 (0.6)	
Observation	220 (22.3)	113 (22.9)	107 (21.7)	
Surgical	12 (1.2)	6 (1.2)	6 (1.2)	
Urgent	748 (75.9)	371 (75.3)	377 (76.5)	
**Hypertension (%)**	277 (28.1)	136 (27.6)	141 (28.6)	0.777
**Diabetes (%)**	305 (30.9)	150 (30.4)	155 (31.4)	0.783
**CKD (%)**	140 (14.2)	69 (14.0)	71 (14.4)	0.927
**Infection (%)**	606 (61.5)	309 (62.7)	297 (60.2)	0.472
**Etiology of liver cirrhosis (%)**				
Viral hepatitis	420 (42.6)	214 (43.4)	206 (41.8)	0.652
Alcohol hepatitis	270 (27.4)	137 (27.8)	133 (27.0)	0.83
Autoimmune hepatitis	21 (2.1)	11 (2.2)	10 (2.0)	1
NAFLD	70 (7.1)	33 (6.7)	37 (7.5)	0.71
**Ascites (%)**	512 (51.9)	253 (51.3)	259 (52.5)	0.75
**HE (%)**	113 (11.5)	51 (10.3)	62 (12.6)	0.317
**GIB (%)**	9 (0.9)	3 (0.6)	6 (1.2)	0.503
**HRS (%)**	52 (5.3)	24 (4.9)	28 (5.7)	0.669
**HCC (%)**	31 (3.1)	15 (3.0)	16 (3.2)	1
**ACLF (%)**	26 (2.6)	13 (2.6)	13 (2.6)	1
**SBP (%)**	70 (7.1)	37 (7.5)	33 (6.7)	0.71
**Other liver failure (%)**	141 (14.3)	64 (13.0)	77 (15.6)	0.275
**Hb (g/dL) (median [IQR])**	10.40 [9.00, 11.90]	10.40 [8.90, 11.90]	10.30 [9.10, 11.70]	0.755
**WBC (10^9/L) (median [IQR])**	5.80 [4.00, 8.47]	5.90 [4.00, 8.30]	5.80 [4.00, 8.60]	0.536
**PLT (10^9/L) (median [IQR])**	91.00 [61.25, 138.00]	93.00 [61.00, 139.00]	88.00 [62.00, 137.00]	0.721
**TBIL (mg/dL) (median [IQR])**	2.60 [1.30, 5.20]	2.50 [1.20, 5.50]	2.70 [1.40, 5.10]	0.406
**ALT (U/L) (median [IQR])**	35.00 [22.00, 59.00]	37.00 [21.00, 61.00]	35.00 [22.00, 55.00]	0.988
**AST (U/L) (median [IQR])**	67.50 [42.00, 113.75]	70.00 [42.00, 119.00]	65.00 [40.00, 110.00]	0.383
**ALB (g/dL) (median [IQR])**	2.90 [2.50, 3.40]	2.90 [2.50, 3.40]	2.90 [2.50, 3.40]	0.955
**ALP (U/L) (median [IQR])**	123.50 [90.00, 178.00]	122.00 [87.00, 176.00]	125.00 [93.00, 180.00]	0.254
**Scr (mg/dL) (median [IQR])**	0.90 [0.70, 1.30]	0.80 [0.70, 1.30]	0.90 [0.70, 1.30]	0.088

**PT (s) (median [IQR])**	17.00 [14.60, 20.30]	17.00 [14.60, 20.30]	17.00 [14.70, 20.30]	0.685
**INR (median [IQR])**	1.60 [1.30, 1.90]	1.60 [1.30, 1.90]	1.60 [1.30, 1.90]	0.991
**Na (mmol/L) (median [IQR])**	136.00 [133.00, 139.00]	136.00 [133.00, 139.00]	137.00 [132.00, 140.00]	0.42
**K (mmol/L) (median [IQR])**	3.90 [3.60, 4.30]	3.90 [3.60, 4.40]	4.00 [3.60, 4.30]	0.479
**MELD (median [IQR])**	17.03 [12.96, 22.29]	17.23 [13.18, 22.20]	16.57 [12.75, 22.53]	0.707
**Ventilation (%)**	15 (1.5)	5 (1.0)	10 (2.0)	0.298
**Liver transplantation (%)**	986 (100.0)	493 (100.0)	493 (100.0)	\
**Hemodialysis (%)**	15 (1.5)	9 (1.8)	6 (1.2)	0.603
**Total dose of rifaximin (mg) (median [IQR])**	\	\	1100.00 [1100.00, 1100.00]	\
**Broad spectrum antibiotic (%)**	164 (16.6)	89 (18.1)	75 (15.2)	0.266
**Human albumin (%)**	435 (44.1)	223 (45.2)	212 (43.0)	0.521
**Vasoactive agent (%)**	8 (0.8)	5 (1.0)	3 (0.6)	0.723

Abbreviations: ICU, intensive care unit; IQR, interquartile range; HE, hepatic encephalopathy; SBP, spontaneous bacterial peritonitis; HRS, hepatorenal syndrome; ACLF, acute-on-chronic liver failure; NALFD, non-alcoholic fatty liver disease; GIB, gastrointestinal bleeding; HCC, hepatocellular carcinoma; Hb, hemoglobin; WBC, white blood cell; PLT, platelet, TBIL, total bilirubin; ALT, alanine aminotransferase; AST, aspartate aminotransferase; ALB, serum albumin; ALP, alkaline phosphatase; Scr, serum creatinine; Na, sodium; K, potassium; PT, prothrombin time; INR, international normalized ratio; MELD, model for end-stage liver disease.
